# Zero-Cycle: First Continuous, Unsupervised, At-Home Monitoring of Spinal Motion as a Per-Cycle Substrate for Machine Learning

**DOI:** 10.3390/s26196102

**Published:** 2026-09-26

**Authors:** Grzegorz Miekisiak, Tomasz Szczepański, Anna Palczewska

**Affiliations:** 1 Vratislavia Medica, Lekarska 1, 51-134 Wrocław, Poland; 2 Clinical Department of Neurosurgery, Center for Neurologic Disorders, 4th Military Hospital, Rudolfa Weigla 5, 50-981 Wrocław, Poland

**Keywords:** continuous monitoring, home monitoring, spinal kinematics, inertial sensing, per-cycle features, drift mitigation, magnetometer-free sensing, cone of economy

## Abstract

**Highlights:**

**What are the main findings?**

- Spinal motion was recorded continuously and unsupervised in the home for 13 to 16 days per patient, yielding 88,027 analysed movement cycles across four patients.
- Patients managed the garment themselves across 87 recording-defined wear sessions, including an 80-year-old, without a clinician present and without a calibration posture at any point.
- The reduction in both surgical patients persisted under every daily summary rule tested; the comparison injection case did not show a comparable reduction despite improvement in patient-reported outcomes.

**What are the implications of the main findings?**

- The output is a uniformly defined, session-comparable per-cycle stream, usable as a substrate for machine learning rather than as a single summary score; no model is trained here.
- Best-200 describes the most compact observed segment of heterogeneous all-day wear, applying the best-of-repeated-efforts convention of clinical measurement to unscripted home life.

**Abstract:**

Continuous, objective records of spinal motion during ordinary life have not been available; assessment relies on intermittent questionnaires and brief supervised motion capture. A dual inertial-sensor garment applies the Zero-Cycle method, which organises the differential thorax–pelvis motion into movement cycles and emits one fixed-dimensional feature vector per cycle, using recurrent movement configurations as body-intrinsic anchors. In the implementation used for this study, the differential orientation is projected onto a horizontal plane at a nominal 50 cm scale, centred on a data-derived hub, and segmented at recurrent radial minima; each cycle contributes a start landmark, an apex landmark and their difference, in centimetres of projected radius. No magnetometer is used, and no calibration posture is required at any point. In a four-patient feasibility pilot conducted unsupervised at home around two foraminotomies and two transforaminal epidural steroid injections, 88,027 movement cycles were analysed over 13 to 16 recorded days per patient, across 87 recording-defined sessions during patient-managed home wear. Because home life is unscripted, the principal daily display is Best-200, the lowest-excursion window of 200 consecutive retained cycles observed that day, read as a candidate estimate of the day’s best-controlled state following the best-of-repeated-efforts convention of standardised clinical measurement; whole-day and alternative summaries are reported alongside. The two surgical patients showed a reduction that persisted under every summary rule (cycle-level Cliff’s delta +0.93 and +0.62); the comparison injection case showed no comparable reduction despite substantial improvement in patient-reported outcomes; the index injection case was summary-dependent. Excursion and return-related quantities are motivated by Dubousset’s cone-of-economy and Panjabi’s neutral-zone concepts, without claiming equivalence to either. The contribution is a substrate for machine learning rather than a more accurate sensor; no model is trained and no treatment effect is established.

## 1. Introduction

Disability and impaired function are hallmarks of spine pathology, but they are assessed clinically only at intervals, through questionnaires, the judgement of an experienced surgeon, and occasional laboratory motion capture, and not as a continuous record of how the spine actually moves during daily life. Such a record does not currently exist. The wearable inertial measurement unit (IMU) is the natural instrument for it, being inexpensive and unobtrusive, but continuous, unsupervised spine monitoring in the home has not been achieved.

What such a record should contain is less obvious than it appears. Spinal control is usually summarised by how far the spine moves, but two distinct descriptions of it already exist in the clinical literature. Dubousset described the cone of economy [1]: the envelope within which the trunk is maintained with least effort, an account of control as economy of excursion. Panjabi described the neutral zone [2,3]: the low-stiffness region about neutral within which the spine offers little resistance to motion, an account of control as the existence of a preferred configuration. The first concerns how far the trunk deviates; the second concerns where it comes back to. Neither has been recorded continuously in a living person during ordinary activity, and they have not been recorded together. In this paper the two concepts motivate two per-cycle quantities, an excursion and a return position; no equivalence to either construct is claimed.

The reasons continuous spine monitoring has been difficult are well known. A recent validation of a spine-specific wearable against optical motion capture attributes the limited clinical adoption of single-sensor IMUs to three requirements: subject-specific calibration, drift correction, and high-fidelity sensor fusion [4], the same problems identified across the inertial-sensing literature [5,6]. Orientation is obtained by integrating angular velocity, so gyroscope bias accumulates as heading drift; the usual correction, magnetometer fusion, is unreliable indoors and near metal, and features computed in the sensor frame shift with each donning and with garment slip, which is why such systems typically require per-subject calibration and degrade outside the laboratory.

A fourth requirement is logically prior to these three and is rarely stated: what quantity should be measured. The three above concern how faithfully a chosen quantity is recovered, and they presuppose that the quantity is range of motion, a joint angle, or a segmental moment—measurands inherited from goniometry and from optical gait laboratories. None of them is defined by the body itself; each requires an external frame to be meaningful, which is what makes calibration and fusion necessary in the first place. The instrument reported here was built to investigate an excursion metric motivated by Dubousset’s cone of economy [1], a construct that is body-referenced rather than laboratory-referenced, and which motivated a differential thoracic-against-pelvic architecture rather than a single-sensor one. The recurrence of a return configuration, cycle after cycle, then provides a second quantity in its own right. The Zero-Cycle programme investigates such recurrent configurations as body-intrinsic anchors for free-living biomechanics; for torso heading, that principle and its long-distance validation are reported in a companion letter [7]. The implementation used for the present clinical recordings is described in full in Section 2; local anchoring of the drift-sensitive heading channel is a programme-level result carried by the companion letter, and no drift bound is claimed for the clinical features reported here beyond what Section 2 establishes.

It is worth stating the novelty precisely, because several adjacent literatures already exist. Continuous home-based movement monitoring is established in neurological and rehabilitation settings, where wearable inertial systems track motor symptoms and physical activity over extended periods [8]. Spine-specific inertial work has largely addressed different problems: validation of trunk kinematics during supervised or standardised functional tasks [4,9,10,11,12], automated spine-angle estimation for telerehabilitation and prompted exercise [13], and the repeatability of spinal-posture measurement in daily life [14]. These studies establish that spine kinematics can be measured with IMUs and that home use is attractive, but none provides a continuous, unsupervised, at-home stream of spine-specific motion sampled at the level of individual movement cycles across an intervention course. The present claim is, therefore, deliberately narrow: not that home wearables, posture sensors, or spine IMUs are new, but that continuous, unsupervised, at-home, per-cycle monitoring of spinal motion, demonstrated through an intervention course in entirely unscripted home life, is the specific paradigm reported here.

The substance of the contribution is what this recording is: a substrate for machine learning. Each movement cycle yields a fixed-dimensional sample with a defined boundary, an activity context and a timestamp. The per-cycle stream is uniformly defined by a single frozen extractor, is produced without labels, and retains the order and heterogeneity of the day’s movement that a session average discards; sparse labels—clinical timepoints, patient-initiated events, longer-term outcomes—attach at the timestamp. Whether these properties translate into transferable learned models is a prospective claim: no model is trained and no cross-subject generalisation is tested in this work.

The contributions are, in order: (i) the first continuous, unsupervised, multi-week recording of spinal motion in the home; (ii) its formulation as a per-cycle machine-learning substrate $X\in R^{N\times d}$, which is the principal contribution; (iii) the explicitly defined per-cycle measurement that produces it, magnetometer-free and requiring no calibration posture; and (iv) a four-patient feasibility pilot showing that the stream can be acquired over weeks of patient-managed wear and shows intervention-associated temporal change, including a within-study discordant comparison case. More broadly, the principle is not specific to the spine: we propose that a natural computational unit of free-living biomechanics is the biomechanically anchored movement cycle rather than the timestamped sample or the session average, and the same construction is applicable in principle to the knee, the hip, or gait as a whole. A phenotype model is not developed here.

## 2. Materials and Methods

### 2.1. Instrumentation

Two BNO085 sensors [15] were worn over the upper thoracic spine and the sacrum (approximately S1), about 50 cm apart, in a soft garment intended for continuous home wear (Figure 1). The garment illustration labels the upper pocket T1; the sensor lies over the upper thoracic region, approximately T1 to T4, and is denoted T throughout. The two-sensor differential configuration was adopted so that trunk excursion could be expressed against the pelvis rather than against a room, following Dubousset’s cone of economy [1]. Each sensor operated in a gyroscope-plus-accelerometer (six-axis) fusion mode with the magnetometer disabled. The sensors cost on the order of a few euros each.

The acquisition firmware writes paired sensor quaternions, linear accelerations and angular velocities to 92-byte packets with a shared packet timestamp, so the two streams share one time base and no inter-sensor clock offset accumulates. The nominal reporting rate is 100 Hz; the analysis software estimates the effective rate per file from the median positive timestamp difference, which evaluated to 90.91 Hz for 1041 of 1042 files and 100 Hz for one. Inter-sensor report latency, packet loss and timestamp-gap handling were not quantified from the raw recordings and are listed in Limitations.

### 2.2. Differential Orientation and the Projected Per-Cycle Representation

This section describes the exact extractor that produced every clinical number in this paper (TARS+ADL-10.py; identifying hash in Supplementary Section S1), correcting the processing description, units and derived interpretation given in the submitted version. Reset-governed feature extraction remains separate, unpublished work outside the scope of this paper, from which no result is drawn. A single frozen version of this extractor was used for every file in the study.

#### 2.2.1. Differential Orientation

Let $q_{S}\left(t\right)$ and $q_{T}\left(t\right)$ be the normalised sacral and thoracic quaternions (scalar-first Hamilton convention [16]). The relative quaternion and its per-file initial reference are

(1) $$q_{d}\left(t\right)=q_{S}{\left(t\right)}^{-1}⊗q_{T}\left(t\right),\;q_{0}={\bar{q}}_{S}^{\;-1}⊗{\bar{q}}_{T},$$

where each $\bar{q}$ is the normalised componentwise mean of the first 100 quaternions of the file. Whole-body turning, common to both sensors, cancels in the differential.

#### 2.2.2. Projection

The extractor forms $q_{m}\left(t\right)=q_{d}\left(t\right)⊗q_{0}^{-1}$ and rotates the unit vector $e_{-}={\left(0,0,-1\right)}^{⊤}$; the horizontal projection is

(2) $$p\left(t\right)=-L\;P_{xy}\;R\;\left(q_{m}\left(t\right)\right)e_{-},\;L=50\;\mathrm{cm},$$

with $P_{xy}$ selecting the first two components, followed by a five-sample median filter per coordinate and a per-file in-plane rotation to a data-derived principal direction (Supplementary Section S2). For a pure tilt θ from the initial vertical, the uncentred magnitude is L|sinθ|, and a pure rotation about the reference vertical leaves the projection unchanged, so axial rotation is not represented in the exported features. *L* is a nominal model scale, not an anthropometric measurement, and the features are not vertebral displacements. All clinical values are reported in the centimetres in which they were computed; after subtraction of the data-derived hub below, no single inverse-trigonometric conversion to an angle exists (verified synthetically in Supplementary Section S3).

#### 2.2.3. Activity Selection, Hub, and Cycle Detection

Combined gyroscope angular-speed magnitude over 3 s windows (1 s step) above 0.15 rad/s marks activity; contiguous active segments shorter than 5 s are dropped, and the retained segments are concatenated for extraction when they exceed 60 s and are less than 90% of the file; otherwise, the whole file is used. The hub h is the weighted centroid of the above-95th-percentile bins of a Gaussian-smoothed 60×60 histogram of the selected coordinates, and the radial signal is $r\left(t\right)=∥p_{\mathrm{processed}}{\left(t\right)-h∥}_{2}$. The radius is Gaussian-smoothed (σ≈0.2 s); a noise scale is 1.4826× the median absolute deviation of the lowest radius quartile, floored at 0.3 cm; boundaries are smoothed-radius minima separated by at least 0.4 s with prominence at least twice the noise scale and a cycle is retained when its duration lies in [0.3,10] s, it contains at least three samples, and its smoothed apex exceeds the larger endpoint radius by three noise scales. This is a motion-energy gate with no posture restriction: cycles arise from any movement, not only gait, and the exported stream is a movement-cycle stream.

#### 2.2.4. Per-Cycle Landmarks

With $a_{n}$, $b_{n}$ the retained boundaries and $k_{n}$ the index of the maximum smoothed radius between them, the exported landmarks are sampled from the less-smoothed radius,

(3) $$v_{n}=r\left(a_{n}\right),\;p_{n}=r\left(k_{n}\right),\;g_{n}=p_{n}-v_{n},$$

the start (return-position) landmark, the apex (excursion) landmark, and their difference. Because $k_{n}$ is selected on the smoothed signal, $g_{n}$ can be negative; such cycles are retained rather than removed (Supplementary Section S3, check 4). Endpoint landmarks contain genuine movement and cannot serve as estimates of sensor error. Every retained cycle carries the software flag validated; the flag records passage of the duration and prominence criteria, not an external validation. The extractor also maintains a diagnostic running quaternion reference, updated at each retained cycle start by short-arc spherical interpolation; it is computed after the projection, hub and segmentation and is not fed back into any exported feature, so no reset-mediated drift bound or frame invariance is claimed for these outputs. A 9 to 32% “return accessibility” figure in the submitted version originated from a separate exploratory analysis outside this extractor and is withdrawn.

### 2.3. The Per-Cycle Feature Vector and the Substrate Stream

Assembled per cycle and stacked over a recording, the landmarks define the substrate object,

(4) $$x_{n}={\left[\;v_{n},\;p_{n},\;g_{n},\;d_{n}\;\right]}^{⊤}\in R^{d},\;X={\left[\;x_{1}\cdots x_{N}\;\right]}^{⊤}\in R^{N\times d},$$

with $d_{n}$ the cycle duration. Each row is one movement cycle; rows are ordered by source file and acquisition order and carry their date, source file and row index. The per-cycle boundary is the natural segmentation marker. *X*, not the sensor, is the deliverable of this work. For reference: the *Zero-Cycle anchor* is the recurrent configuration at a detected boundary; the *Zero-Cycle token* is the fixed-dimensional feature vector emitted for one cycle; the *Zero-Cycle substrate* is the ordered sequence *X* with its timestamps, quality indicators and extractor version; and *biomechanical tokenisation* denotes the conversion of a continuous wearable recording into this sequence of movement-defined units.

### 2.4. Suitability as a Machine-Learning Substrate

Four properties motivate *X* as a learning input; they are structural properties of the representation, and their value for learning is a prospective claim.

*Uniform tokenisation.* Fixed-dimensional rows with naturally detected boundaries correspond to the input sequence models expect [17]; imperfect returns enter the sequence as informative tokens rather than being dropped.

*Consistent definition across sessions.* All features are computed by one frozen extractor under one fixed set of constants, so a value on one day is defined identically to a value on another. Whether the features are additionally invariant to placement change on the body is a separate empirical question that the wear-session data motivate but do not settle (Section 3.6).

*Label-free production.* The stream is produced without clinical labels; timepoints, patient-initiated pain events and longer-term outcomes attach at the timestamp, supporting self-supervised pretraining with light supervision [18,19].

*Version-pinned reproducibility.* Features are extractor-version-dependent, so the version is pinned, its hash reported, and versions never mixed across comparisons; every table and figure in this paper is reproducible from the released exports and scripts.

### 2.5. Cohort and Protocol

Four patients were monitored. The index patient (Z) was an 80-year-old with severe L3/L4 sciatica treated with transforaminal epidural steroid injection (TESI). A second TESI patient (P) presented with sciatica for which MRI showed no corresponding lesion, and serves as a within-study discordant comparison case. Two further patients (F and K) underwent single-level foraminotomy. Each patient wore the device continuously in their own home, unsupervised: Z on 13 recorded days (4–18 February 2026), K on 16 (9–26 March), P on 16 (10–25 March), and F on 16 (14–29 April). A recorded day does not imply uninterrupted 24-h wear. Intervention dates from the clinical record: TESI 5 February (Z) and 12 March (P); foraminotomy 10 March (K) and 15 April (F). Patient-reported outcomes were recorded on the first and last day of each monitoring period using the Oswestry Disability Index (ODI) [20] and the Core Outcome Measures Index (COMI).

The garment was fitted once, at the start of monitoring, and thereafter handled entirely by the patient. Segmenting each recording at interruptions longer than two hours yields 87 recording-defined wear sessions across the cohort (Section 3.6).

### 2.6. Analysis

The unit of analysis was the movement cycle. Recording days yielding fewer than 200 cycles cannot supply the 200-cycle daily metric defined below; four such days were excluded from count-matched comparisons (they remain in the daily record), leaving 88,027 analysed cycles (Z 19,133; K 38,210; P 17,959; F 12,725). The clinical sample is four patients, and the cycle is a descriptive unit within each patient rather than an independent observation; because adjacent cycles are serially correlated and nested within bouts, days and patients, no population-level significance test is appropriate and none is reported.

#### 2.6.1. Best-200: Rationale and Definition

For day-to-day comparison, the per-cycle stream is summarised with a fixed-size window rather than a whole-day average, and the rationale deserves stating in full because this is, to our knowledge, the first spinal-motion study conducted entirely in unscripted home life. Supervised studies obtain day-to-day comparability by prescribing the task: every session is the same walk, the same bend, the same test, so a session mean compares like with like. That option does not exist here by design. A patient’s day contains carrying, stairs, hurrying, turning in a kitchen and slow ambling, in proportions that differ every day, and a whole-day median, therefore, compares mixtures of behaviour, not states of the spine. Best-200 seeks comparability from the data rather than from a protocol: the same selection rule is applied to each day, identifying its lowest-median-apex window of 200 consecutive retained cycles, and these windows are compared across days. The rule gives consistent selection; the selected windows are not assumed to contain identical activities. Demanding activity can widen the observed excursion for entirely healthy reasons. The minimum-window summary is intended to reduce the influence of demanding activities on the daily readout, while remaining sensitive to the activities and recording conditions available that day. Best-200 is, therefore, read operationally as the lowest-excursion window of 200 consecutive retained cycles the day contained, and interpreted as a candidate estimate of the day’s best-controlled state, an interpretation that activity context, sampling conditions and hub estimation can also influence. Reporting the best of repeated efforts rather than their average is an established convention in clinical measurement: standardised spirometry takes the best of at least three acceptable manoeuvres as the endpoint, because effort and context vary while capacity does not [21]. The analogy is one of measurement philosophy, motivating the choice of summary; it does not confer spirometry’s validation on this metric.

Formally, for *M* cycles in acquisition order and window starts j=1,…,M−199, the selected window minimises the median apex,

(5) $$j^{*}=\arg\min_{j}\mathrm{median}\left\{p_{j},\ldots ,p_{j+199}\right\},$$

with ties resolved to the earliest start; the daily Best-200 values are the median apex and median start of that window, and error bars are its interquartile range. Windows may cross file boundaries and are not walking bouts. As with any observed minimum, the value depends on the candidate windows the day provides and can only stay the same or decrease as more of the recording is included; Section 3.7 quantifies this on nested prefixes and reports the whole-day median, first-200 and a second minimisation rule alongside, because the summaries answer different questions and none substitutes for another.

#### 2.6.2. Within-Patient Effect Sizes and Uncertainty

Cliff’s delta [22] is computed at the cycle level between explicitly defined earlier and later calendar windows (Table 1), oriented so that a positive value indicates lower later values,

(6) $$\delta =\frac{\#\left\{x_{i}^{E}>x_{j}^{L}\right\}-\#\left\{x_{i}^{E}<x_{j}^{L}\right\}}{n_{E}\;n_{L}}.$$

Because cycles are serially correlated, uncertainty is expressed by 2000 whole-day bootstrap resamples drawn independently within the earlier and later windows; where the earlier window is a single day, that day is fixed in every resample and the interval understates baseline uncertainty, which is stated with the values. Z’s mixed treatment date (5 February) is retained in the daily trajectory and omitted from the two-window comparison.

### 2.7. Validation

Implementation behaviour, event identity, orientation and feature accuracy, mounting repeatability, drift behaviour and clinical interpretation are separate questions and are evaluated separately. Synthetic checks of the documented equations (Supplementary Section S3) establish what the computation does; they do not establish accuracy on the body. Concurrent optical motion capture was not used; accuracy of the underlying differential thorax–pelvis orientation, and of the derived projected-radius features, against an external reference is, therefore, not established by this work. A body-referenced optical comparison is feasible and appropriate: optical thoracic orientation can be expressed relative to pelvic orientation and the same projection and event definitions applied to both streams. That experiment is part of the planned work. Long-duration drift behaviour of the heading channel is addressed in the companion letter [7] for the navigation task; no drift bound is claimed for the clinical features reported here, whose local definitions do not accumulate heading increments.

## 3. Results

### 3.1. Overview

Across the four patients, 88,027 movement cycles were analysed over 13 to 16 recorded days each, entirely in the free-living home setting and without a magnetometer. The result is a monitoring result rather than a single task result: one frozen extractor produced an ordered sequence of identically defined cycle-level measurements throughout ordinary home activity, and the clinical course appears as a distributional process—the per-cycle band narrows, widens, fragments, or stabilises over time—rather than as a difference between two scalar values. The full exports contained 88,417 cycles across 61 recorded patient-days; the four dates below the 200-cycle threshold (F 15 April, 101; P 12 March, 102; Z 17–18 February, 10 and 177) remain in the daily record, annotated.

### 3.2. The Per-Cycle Stream

Figure 2 shows the per-cycle stream for the index patient across the monitoring period. In each panel, every movement cycle contributes two values plotted against cycle count: the within-cycle excursion (apex) and the return position (start landmark). The panels run from the pre-treatment baseline through the post-injection course; each contains several hundred to several thousand cycles recorded continuously during unsupervised home wear. The separation between the two bands and the dispersion within each band are the per-cycle quantities summarised in the analyses that follow, and the figure shows that they are defined at the level of individual cycles rather than as session averages. Equivalent plots for the other three patients are in Supplementary Section S4 (Figure S1).

### 3.3. Clinical Course Across the Cohort

Figure 3 summarises the four patients using the Best-200 daily metric: for each recording day, the median apex and median start of the selected 200-cycle window, plotted against day relative to intervention. The two foraminotomy patients (F and K) show a reduction in the per-cycle metric around the intervention that is then maintained. The index TESI patient (Z) shows a reduction under this display with later re-widening; as Section 3.7 shows, Z’s direction depends on the summary rule and a uniformly sustained reduction is not supported for this patient. The comparison TESI patient (P), whose sciatica had no corresponding lesion on MRI, shows no such reduction.

Two recordings show a late partial re-widening. The descriptive activity outputs (Supplementary Section S5, Figure S2) indicate differing activity contexts around it, with falling walking volume in patient Z and the highest walking volumes of the recording in patient K; because these activity estimates are heuristic and not independently validated, they are reported as contextual observations only and are not used to infer mechanism. Candidate clinical explanations, a concurrent hip process or waning of the injection effect in Z and a return to unrestricted activity in K, cannot be distinguished by this design.

### 3.4. Intervention-Associated Change and Effect Sizes

Table 2 quantifies the trajectories as per-patient cycle-level effect sizes over the calendar windows of Table 1, with day-resampling intervals. Both surgical patients are positive on every channel; the comparison case P is negative on every channel with the gap interval crossing zero; the index patient Z is positive but small in the cycle-pooled comparison. The effect sizes reported in the submitted version for Z (+0.87) and P (−0.29) were not reproducible from the archived exports under any window definition tried and are replaced by the values below.

The structural classification is radiological rather than symptomatic. Both TESI patients presented with sciatica; they differ in that the index patient had lateral recess stenosis at the symptomatic level on MRI before treatment (Figure 4), whereas patient P had no corresponding lesion. That distinction, and not the presenting complaint, is what separates them.

As an example of the underlying channels, patient K’s whole-day start median fell from 14.9 cm on the pre-operative day to a later median of about 2 cm and the whole-day apex median from 48.6 cm to 9.1 cm; the pre-operative day was heterogeneous, with two source files contributing most of its high-radius cycles, so the magnitude (though not the direction) of K’s change depends on how that day is represented (Supplementary Table S3).

### 3.5. Relation to Patient-Reported Outcomes

Patient-reported outcomes for the same monitoring periods are given in Table 3. All four patients reported large improvement on both instruments: ODI fell by 34 to 56 percentage points and COMI by 2.4 to 6.7 points, and every patient moved from the severe or worse category to minimal or moderate disability. The per-cycle measurement agrees with that improvement in the two surgical patients, is summary-dependent in the index patient, and shows no comparable reduction in the comparison case P despite substantial improvement in patient-reported outcomes. The instrument and the questionnaires, therefore, separate the one patient in whom no structural lesion was identified. A single case is an observation and not a rate; it is reported because the capacity to disagree with a questionnaire is the property that would make continuous measurement useful. This discordance is exploratory and does not establish which measure better reflects the clinical course or whether the movement features provide incremental clinical information.

### 3.6. Unsupervised Wear and Self-Refitting

The garment was fitted once, at the start of monitoring, and thereafter managed entirely by the patient, without supervised refitting and without an instructed calibration posture at any point in the protocol. Segmenting each recording at interruptions longer than two hours yielded 87 recording-defined wear sessions (Z 11, K 16, P 15, F 45). Because recording interruptions were not independently annotated as removal and replacement of the garment, this count is not a verified count of redonning events. F used the garment in short repeated bouts, three sessions on a typical day; Z, K and P wore it in long blocks. The index patient was 80 years old. No session was excluded on placement grounds. These sessions document the feasibility of patient-managed wear—the fitting appointment that confines wearable musculoskeletal measurement to supervised sessions was not required—while between-session placement offsets and mounting repeatability were not quantified, so mounting invariance is not established and is a defined target of the planned optical study.

### 3.7. Sensitivity of the Daily Summary

Every count-matched patient-day was recomputed under four rules: whole-day median, Best-200 (Equation (5)), first-200, and a second minimisation rule using the apex-minus-start difference; full values are in Supplementary Table S1 and Figure S3. The reduction in the two surgical patients and the absence of reduction in the comparison case are preserved under every rule. In the index patient, the direction depends on the rule: whole-day and both minimum-window summaries fall, while the first-200 summary rises, so a uniformly sustained reduction is not supported for Z, and the scalar exports cannot distinguish early-day activity differences, recording conditions, artefact, or genuine distributional change as the cause.

The opportunity dependence of Best-200 was quantified directly. Across the fourteen days containing at least 2000 cycles, the median Best-200 apex was 14.79 cm when each day was restricted to its first 250 cycles, 10.60 cm at 500, 8.33 cm at 1000 and 7.08 cm at 2000 (Supplementary Figure S4 and Table S2): the expected monotone behaviour of an observed minimum as more of the recording is included. The comparison quantifies opportunity dependence; it does not distinguish additional sampling opportunity from changes in the activities represented, nor establish a physiological lower bound. The ratio of median daily cycle counts in the later versus earlier windows was 0.89 in K, 0.90 in P, 1.98 in Z and 3.18 in F. All else being equal, these ratios describe differences in sampling opportunity; they do not by themselves establish the amount or direction of bias when activity composition also changes. The dependence acts against the observed reduction in K and P and in favour of it in F, whose single pre-operative day offered 294 cycles, which is why F’s whole-day comparison—involving no window selection and showing the same reduction (15.3 to 4.2 cm)—is reported alongside.

## 4. Discussion

The principal contribution is the per-cycle representation rather than the sensor or its accuracy: a uniformly defined description of spine movement obtained over weeks in the home rather than in a brief supervised session. The submitted version attributed the feasibility of this description to a per-cycle reset acting on the features; the extractor that produced these data computes hub-centred projected radii and its running reference is diagnostic only, so that attribution is corrected here, and the observation stands on its own: the representation can be computed from extended, patient-managed home recordings, and some trajectories show substantial distributional change.

Using the cycle as the unit of representation is suited to sequence models: regularly ordered, fixed-dimensional samples with a defined boundary correspond to the inputs such models expect [17], with the per-cycle boundary as the segmentation marker. Retaining cycles with imperfect returns, and recording the return position, preserves information a reset-only pipeline would discard; the distribution of return positions is itself a feature. The representation is produced without labels, which supports label-efficient training [18,19], with sparse labels applied at the corresponding timestamps; no model is trained here, and neighbouring cycles must not be treated as independent patients in any future validation.

Best-200 addresses the distinction between the most compact observed segment and the average of everything the patient happened to do. The patient is not performing a laboratory manoeuvre throughout the day: a larger whole-day distribution may reflect a broader activity repertoire or more demanding movement as well as a change in control, so replacing Best-200 with a whole-day median would change the measurement question rather than remove bias, for the same reason spirometry reports the best acceptable manoeuvre rather than the average of all attempts [21]. At the same time, the selected minimum depends on the opportunities the record provides, which the nested-prefix analysis quantifies, and divergence between Best-200 and whole-day trajectories—as in patient Z—is reported and examined rather than interpreted as confirmation or failure of either.

Monitoring of this kind is likely to be most informative where it disagrees with questionnaire scores, and discordance with patient-reported measures is of interest rather than a limitation of the instrument. The present cohort contains one such case: patient P reported substantial improvement on both instruments, moving from severe to minimal disability, while the per-cycle measurement showed no comparable reduction. Which of the two is the better guide to that patient’s course cannot be settled here and requires longer follow-up; a measurement can disagree with a questionnaire because it captures another domain, because its sampling context changed, or because it is inaccurate, and agreement would not imply that it adds nothing. What the case establishes, at most, is that the two do not measure the same thing, which is the precondition for the measurement adding anything.

The two per-cycle quantities correspond to the two established descriptions of spinal control that motivated the instrument. The excursion metric is motivated by Dubousset’s cone of economy [1], measured here once per cycle rather than as a static posture; the return-position metric is potentially related to Panjabi’s neutral-zone concept [2,3], which was defined from cadaveric segmental testing under applied load [23]. The present quantities are neither measurements of balance effort nor of segmental stiffness; both correspondences are offered as interpretation, establishing them would require concurrent reference standards not collected here, and whether the two quantities carry independent clinical information is a question for a cohort rather than for four patients. These interpretations motivate the quantities but are not required for the substrate argument, which rests only on the per-cycle stream being uniformly defined, prolonged and informative.

This representation is intended as the input layer for downstream tasks: movement phenotyping, prediction of symptom flares and longer-term outcomes, and automated generation of structured progress notes from the per-cycle stream. Because the recording is continuous and unsupervised, the same stream supplies behavioural context in which the kinematic features are read; the extractor’s heuristic activity outputs are descriptive only (Supplementary Section S5), and validated activity recognition from the same sensors [18,24] is the subject of concurrent work. A single-sensor configuration is also possible in principle, without the differential inter-segmental signal that two sensors provide.

## 5. Limitations

This is a pilot of four patients, and all comparisons are within-subject across days; there is no untreated control group, randomisation or blinding, and no inference about a population is drawn. Four patients do not usefully estimate the between-patient variance of the pre-to-post change, so no power calculation is offered; the per-patient effect magnitudes are reported partly to serve as input to that calculation for the confirmatory study, which is designed with several days of monitoring on both sides of the intervention and prespecified endpoints.

The pre-intervention window rests on a single recording day in three patients, so each effect size compares one baseline day against a multi-day later window and baseline day-to-day variability is uncharacterised; whole-day bootstrapping cannot create unobserved days. This asymmetry is partly structural to the clinical setting: the interval between the decision to treat and the intervention is set by symptom severity, and the pre-intervention period is also the period of lowest ambulatory volume. The conventional pre-operative baseline in spine surgery is a single administration of a questionnaire; the single recorded day nonetheless remains a weaker baseline than the multi-day later window it is compared against, and the effect sizes should be read accordingly.

The measurand is a projected, hub-centred radial signal in centimetres at a nominal 50 cm scale: not vertebral displacement, not a complete three-dimensional orientation (axial rotation is not represented), not segmental stiffness or load. First-sample initialisation, hub dependence on the recorded motion distribution, filtering across concatenated intervals and file-boundary reinitialisation may affect comparisons. No concurrent optical reference was collected, so the accuracy of the underlying differential orientation and of the derived projected-radius features against an external reference is unestablished; the body-referenced optical comparison described in Section 2.7 is planned. The diagnostic reference tracker is not connected to the exported features, so this study establishes neither reset-mediated drift suppression nor mounting invariance, and unsupervised wear without a calibration pose does not by itself demonstrate robustness to placement or slip; the 87 recording-defined sessions document feasibility only.

Event identity was not externally validated: movement cycles can arise during activity other than gait, and no posture restriction excludes seated or recumbent movement from extraction. The instrumented segment spans approximately T1 to S1; the cervical spine is not monitored and individual vertebral segments are not resolved, and sustained static loading during sitting, a major contributor to spinal load, is not quantified by this representation. The activity estimates were not independently validated and are used only as descriptive context, not to establish a clinical mechanism or the independence of the kinematic features from activity exposure. Best-200 is an observed extremum whose estimate depends on candidate-window opportunity (quantified in Section 3.7); days with fewer than 200 cycles cannot supply it. Negative apex-minus-start values are an expected property of the landmark definitions and are retained. The neutral-zone and cone-of-economy interpretations remain unvalidated, and no normal range, threshold or minimal clinically important difference is established.

## 6. Conclusions

We have presented the first continuous, unsupervised, multi-week recording of spinal motion in the home, and its principal property: a per-cycle stream suitable as a substrate for machine learning. A single frozen, fully specified extractor produced, for every movement cycle of 87 recording-defined sessions of patient-managed wear, a fixed-dimensional feature vector that is identically defined across days and patients, uniformly segmented, and version-pinned. In four patients across two intervention types, the stream was acquired over weeks of unscripted home life and showed intervention-associated temporal change: a reduction in both surgical patients that persisted under every daily summary rule, no comparable reduction in a discordant comparison case despite substantial improvement in patient-reported outcomes, and summary-dependent behaviour in the index case, which is reported as such. The daily Best-200 metric is read as the lowest-excursion window of 200 consecutive retained cycles of each day, following the best-of-repeated-efforts measurement philosophy. The excursion and return-position quantities are motivated by, and not equated with, Dubousset’s cone of economy and Panjabi’s neutral zone. The contribution offered here is the substrate itself and its feasibility in prolonged patient-managed home wear; orientation and feature accuracy against an external reference, mounting repeatability and the value of the representation for learned models are the defined next validation steps, and development of that layer is the subject of future work. 

## Figures and Tables

**Figure 1:**
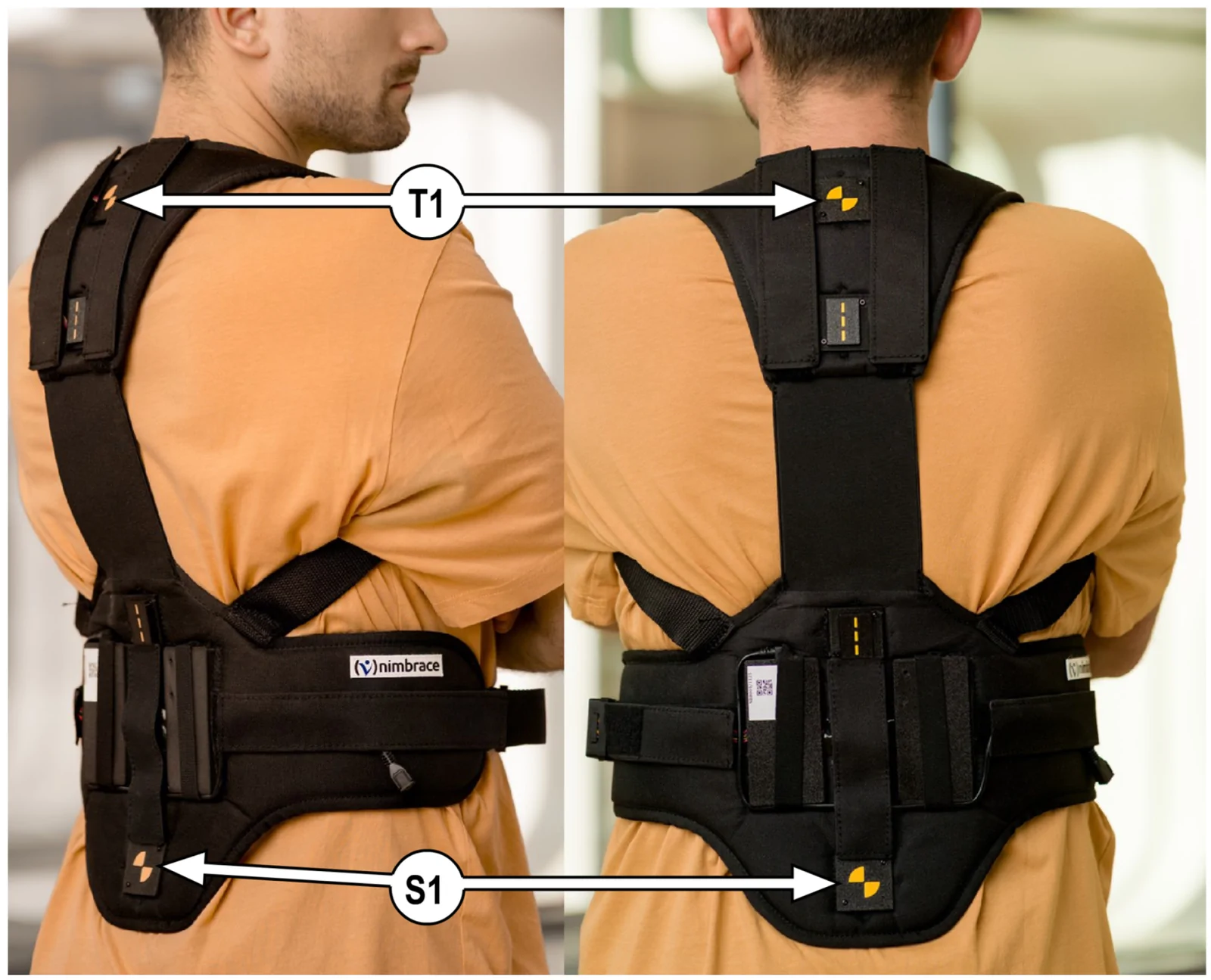
The instrumented garment, shown in lateral (**left**) and posterior (**right**) views. A soft, over-the-shoulder garment houses two inertial sensors, one over the upper thoracic spine and one over the lumbosacral region, each integrated into a fabric pocket so that placement is reproduced approximately at each donning. The differential orientation of the two sensors yields inter-segmental spine motion. The garment is worn continuously during ordinary activity at home; no external reference, marker, or supervised calibration posture is required.

**Figure 2:**
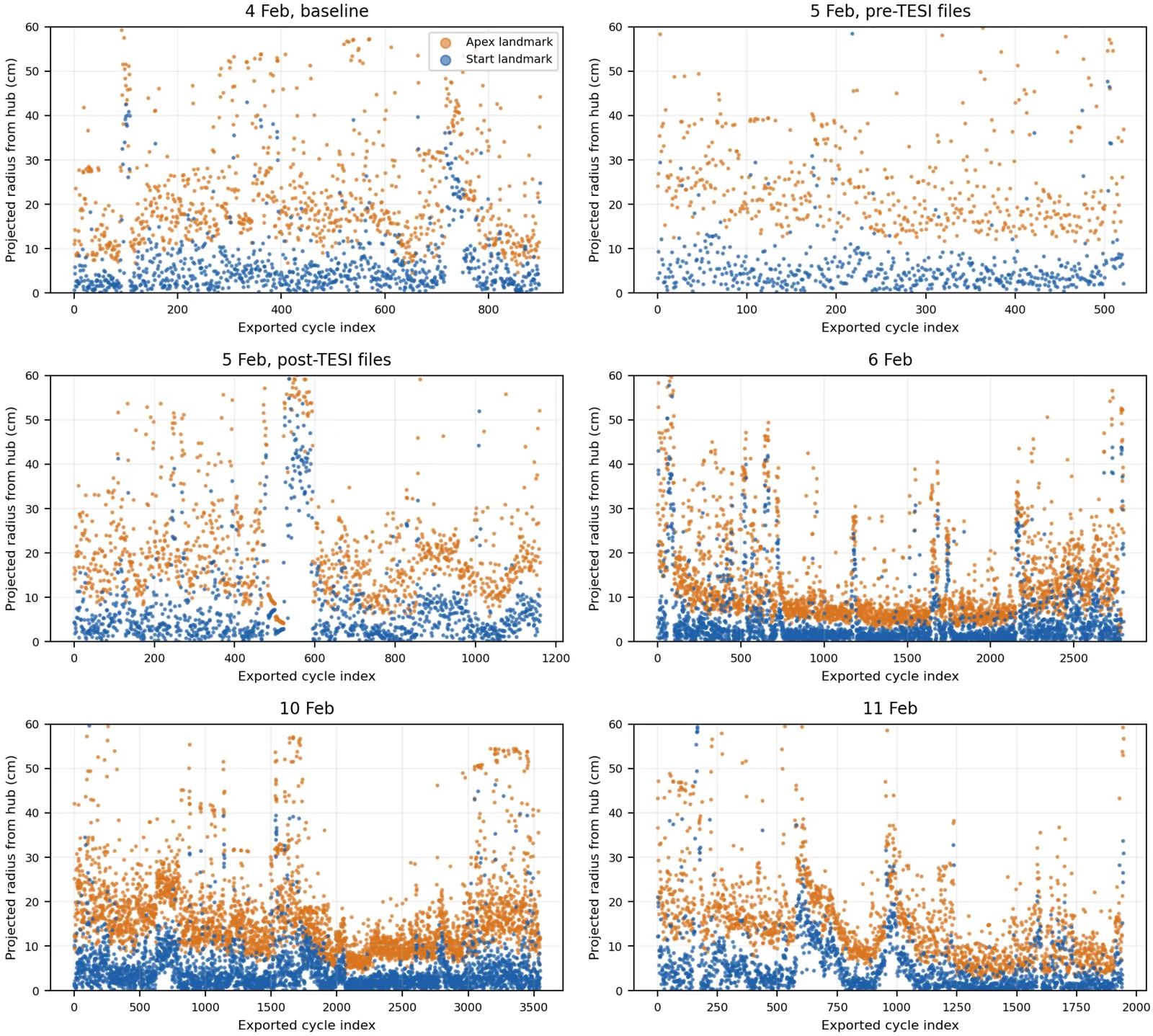
Per-cycle stream for the index patient (Z) across recording sessions, from the baseline day through the post-injection course. For every movement cycle, the excursion (apex, orange) and the return position (start, blue) are plotted against the exported cycle index. Units are centimetres of hub-centred projected radius (Section 2.2).

**Figure 3:**
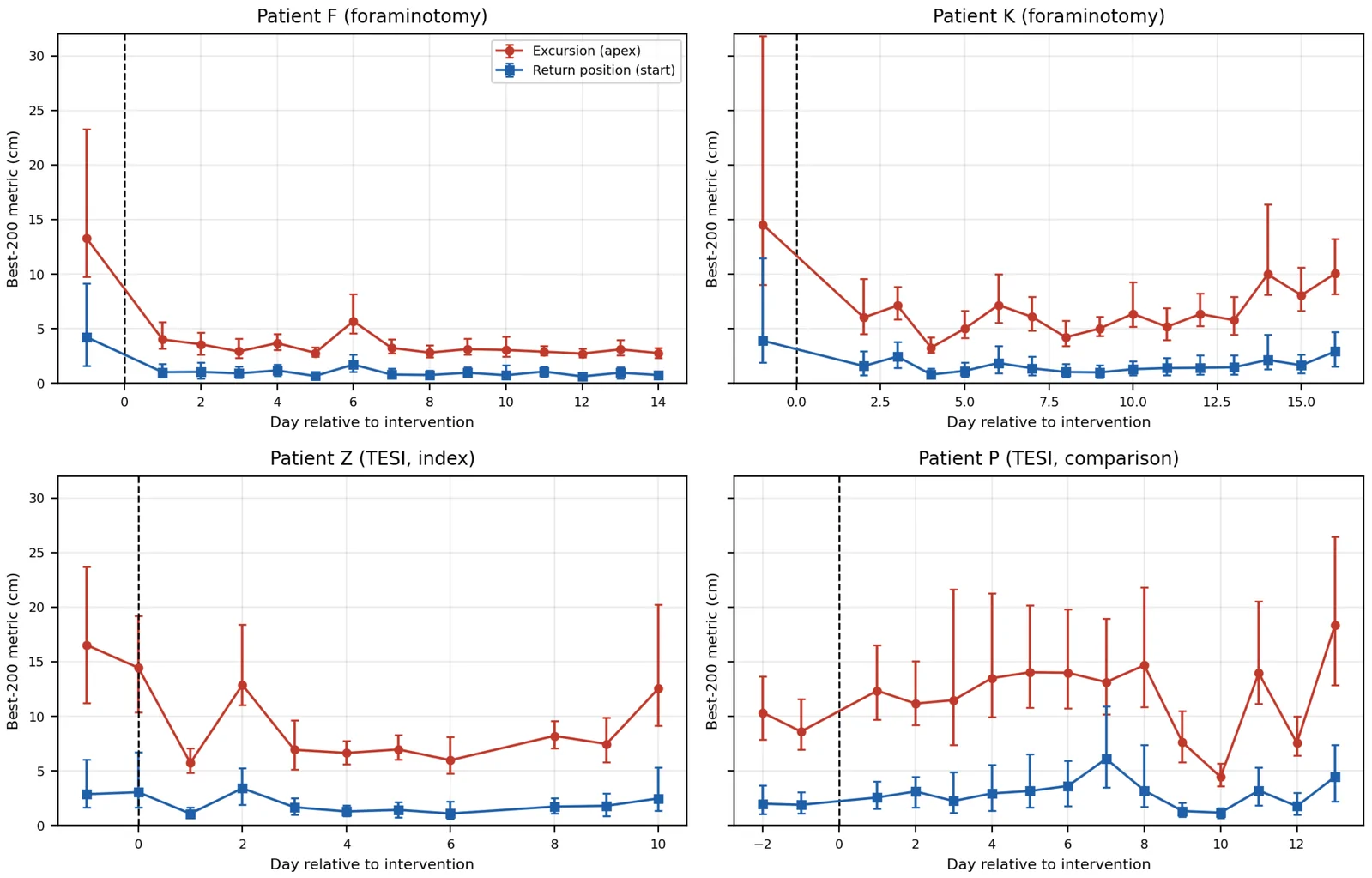
Four-patient Best-200 trajectories. For each patient, the median excursion (apex, red) and median return position (start, blue) of the selected 200-cycle window are plotted against day relative to intervention (dashed line); error bars are the interquartile range within the selected window. Patients F and K underwent foraminotomy; patients Z and P received transforaminal epidural steroid injection. Units are centimetres of the Best-200 metric; common vertical scale. Days with fewer than 200 cycles cannot supply the metric and are absent here but retained in the whole-day record (Supplementary Figure S3).

**Figure 4:**
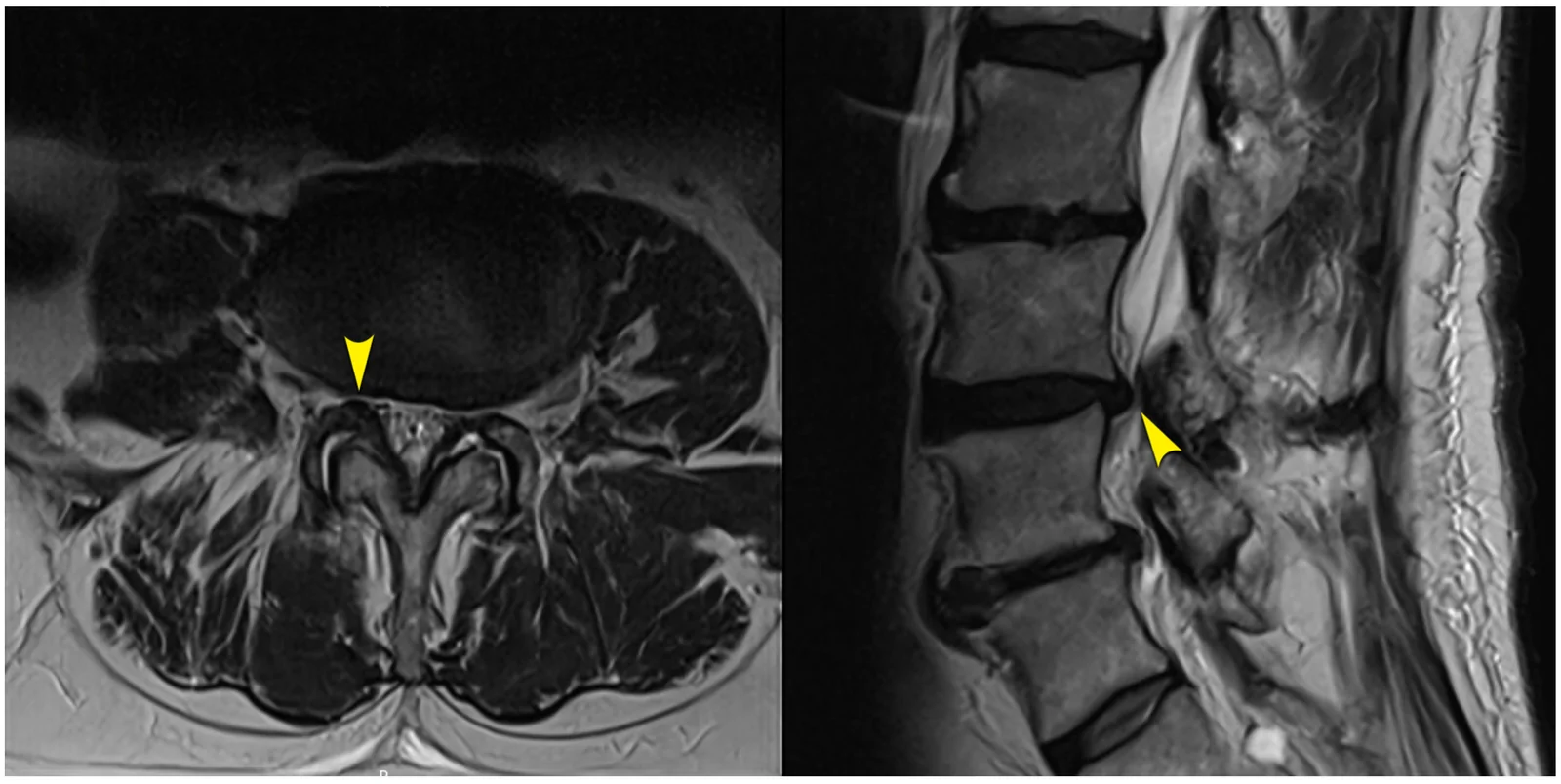
Index patient (Z), T2-weighted lumbar MRI before treatment. Axial (**left**) and sagittal (**right**) images show lateral recess stenosis at the symptomatic level (arrowheads).

**Table 1:** Explicit calendar windows for all earlier-versus-later comparisons. All included dates have at least 200 cycles; Z’s mixed treatment date is omitted from the comparison but not from the trajectory.

Patient	Earlier (2026)	Later (2026)	Days E/L	Cycles E/L
F	14 April	16–29 April	1/14	294/12,431
K	9 March	12–26 March	1/15	2733/35,477
P	10–11 March	13–25 March	2/13	2142/15,817
Z	4 February	6–15 February (available)	1/9	900/16,550

**Table 2:** Per-patient cycle-level Cliff’s delta on the apex-minus-start channel, with conditional 95% whole-day resampling intervals (2000 resamples). A positive value indicates lower later values, not an established treatment effect. With one earlier day (F, K, Z) that day is fixed in every resample, so baseline day-to-day variability is not represented; start- and apex-channel values are in Supplementary Table S1.

Patient	Intervention	Cliff’s δ [95% CI]	Per-Cycle Pattern
Z (index)	TESI	+0.33 [+0.21, +0.44]	Reduction under the Best-200 display with later re-widening; direction summary-dependent (Section 3.7)
P	TESI	−0.18 [−0.37, +0.04]	No comparable reduction; within-study discordant comparison case
F	Foraminotomy	+0.93 [+0.89, +0.95]	Reduction to a tight, low plateau held through follow-up
K	Foraminotomy	+0.62 [+0.56, +0.68]	Reduction with later partial re-widening; heterogeneous baseline day (leave-one-file-out in Supplementary Table S3)

**Table 3:** Patient-reported outcomes at the first and last day of each monitoring period, with the per-cycle effect size from Table 2. ODI, Oswestry Disability Index; COMI, Core Outcome Measures Index.

Patient	ODI (%)	COMI (/10)	Cliff’s δ	Relation
Z (index)	62→14	7.4→2.7	+0.33	Concordant in direction; summary-dependent
K	56→10	4.0→1.6	+0.62	Concordant
F	78→22	9.05→2.35	+0.93	Concordant
P	46→12	6.0→1.4	−0.18	Discordant

## Data Availability

The original data presented in the study are openly available in the Zero-Cycle Spine directory of https://github.com/gmiekisiak/spinerebel (accessed on 8 September 2026), together with the extractor source and its identifying hash (Supplementary Section S1), the analysis pipeline, and the scripts that reproduce every table and figure in this paper.

## Supplementary Materials

The following supporting information can be downloaded at: https://www.mdpi.com/article/10.3390/s26196102/s1, Section S1: Extractor identification and provenance; Section S2: Source-level processing details; Section S3: Synthetic checks of the documented equations (with S3_synthetic_checks.json); Section S4: Per-cycle streams for the remaining patients; Section S5: Secondary activity outputs (descriptive only); Figure S1: Per-cycle start and apex landmarks for patients K (a), F (b) and P (c) over the first six recorded days; Figure S2: Heuristic activity-classifier outputs per recorded day and patient (descriptive only); Figure S3: Daily apex value under four summary rules for every recorded patient-day; Figure S4: Median Best-200 apex on nested prefixes of 250, 500, 1000 and 2000 cycles; Table S1: Daily summaries for every recorded patient-day under the whole-day, first-200, Best-200-apex, Best-200-gap and all-window rules (CSV); Table S2: Best-200 apex per day and nested prefix size (CSV); Table S3: Patient K leave-one-file-out Cliff’s delta (CSV).
