# Supplementary material for: Zero-Cycle: First Continuous, Unsupervised, At-Home Monitoring of Spinal Motion as a Per-Cycle Substrate for Machine Learning
Source: Sensors (Basel). 2026 Sep 26;26(19):6102. doi: 10.3390/s26196102 (PMC13667603; doi:10.3390/s26196102)
Supplement: Supplementary file 1 [file sensors-26-06102-s001.zip › Supplementary File S1.pdf]

Supplementary Material

Zero-Cycle: First Continuous, Unsupervised, At-Home Monitoring of Spinal Motion as a Per-Cycle Substrate for Machine Learning

Miekisiak, Szczepański, Palczewska

S1. Extractor identification and provenance

The clinical exports were produced by the extractor `TARS+ADL-10.py`, which identifies itself internally as TARS-31/Zero-Anchor v10.

File	`TARS+ADL-10.py`
Internal version string	TARS-31/Zero-Anchor v10
SHA-256	`cb53bd29d7ad6a1993f5e9e733a931c8bc6eb0272d2a0f3c96a72fed478c7e76`
Language / dependencies	Python 3; numpy, scipy

The archived cycle-level CSV exports do not embed a build hash or configuration record. Build-to-export provenance therefore rests on the export schema matching the inspected source, which it does: every exported column is produced by the inspected code, and several field definitions (the `validated` flag, the legacy `amplitude` field, the diagnostic tilt and quaternion-difference channels) are explained only by it. This limitation is stated in Section 2.2 of the main text. The source file, the archived exports, and every reanalysis script used in this revision are released together (Data Availability Statement), so all tables and figures are reproducible from the released materials alone.

S2. Source-level processing details

This section records implementation details referenced from Section 2.2 of the main text. The released source is the authority; the values below are transcribed from it.

Coordinate processing. Each horizontal projection coordinate (Equation 2) is median-filtered with a five-sample kernel before hub estimation. A data-derived in-plane rotation is then applied: the principal direction of the projected coordinates over the selected dynamic intervals is estimated by eigen-decomposition of their covariance, and the coordinates are rotated so that this principal direction is aligned to a fixed axis. The rotation is estimated per file, from the same selected intervals that feed the hub histogram.

Activity gate. Combined angular-speed magnitude from the two gyroscopes is averaged over 3 s windows advanced in 1 s steps; windows exceeding 0.15 rad/s are active. Contiguous active segments shorter than 5 s are discarded. If retained active time exceeds 60 s and is below 90 % of file duration, the retained segments are concatenated for extraction; otherwise the whole file is used. Concatenation joins non-adjacent intervals before smoothing; effects at the joins were not quantified from the exports.

Hub. A 60×60 two-dimensional histogram of the selected, processed coordinates is smoothed with a Gaussian of two-bin standard deviation; the hub is the weighted centroid of the bin centres whose smoothed counts exceed the 95th percentile.

Cycle detection constants. Radius smoothing, Gaussian $\sigma \approx 0.2\,\mathrm{s}$ (implemented as a sample count from the estimated rate, rounded to an integer). Noise scale, 1.4826× the median absolute deviation of samples in the lowest radius quartile, floored at 0.3 cm; if fewer than ten such samples exist, first differences are used as a fallback. Boundary candidates, minima of the smoothed radius separated by at least 0.4 s (integer sample conversion) with prominence at least 2× the noise scale. Cycle retention, duration 0.3 to 10 s, at least three samples, smoothed apex exceeding the larger endpoint radius by at least 3× the noise scale. Landmarks are sampled from the less-smoothed radius at the retained indices.

Reference initialisation. The per-file initial reference quaternions are the normalised componentwise means of the first 100 samples of each stream, without sign alignment. The diagnostic running reference is updated at each retained cycle start by short-arc spherical interpolation toward the observed relative quaternion with weight 0.10; it is not fed back into any exported feature.

Timing. The per-file sample rate is the reciprocal of the median positive packet-timestamp difference; 1,041 of 1,042 files evaluated to 90.91 Hz and one to 100 Hz. Cycle durations are sample counts divided by this estimated rate; cycle start times within the file are computed internally but are not exported.

S3. Synthetic checks of the documented equations

The checks below were run on an independent implementation of the equations of Section 2.2, written for this supplement from the documented definitions. They establish what the documented computation does; they do not establish accuracy on the body. Numerical outputs are in `S3_synthetic_checks.json`.

Check 1: pure tilt. A relative rotation of pure tilt θ about a horizontal axis, applied to Equation 2 with $L = 50\,\mathrm{cm}$, yields an uncentred projection magnitude of $L\sin\theta$ at machine precision for $\theta \in \{5, 15, 30, 45, 60\}^\circ$ (e.g. $30^\circ \to 25.000\,\mathrm{cm}$). This confirms the sine, not tangent and not linear-angle, relation stated in Section 2.2.

Check 2: rotation about the reference vertical. Pure rotations of 10° to 170° about the reference vertical produce a projection change of exactly zero. Axial rotation is invisible to the exported features, as stated.

Check 3: no angular recovery after hub subtraction. Two orientations of equal tilt about different horizontal axes, with the hub placed at their centroid, give identical hub-centred radii while representing different orientations. No single inverse-trigonometric map from the exported radius to an angle exists, as stated in Section 2.2.

Check 4: smoothed-apex landmark. On a synthetic radius trace with a brief noise burst, the apex landmark (raw radius at the smoothed-signal argmax) is below the raw maximum, and start/apex landmarks can produce a negative apex-minus-start value. Negative g_n values in the exports are therefore an expected property of the definitions, not corruption; they are retained, as stated in Section 2.2.

S4. Per-cycle streams for the remaining patients

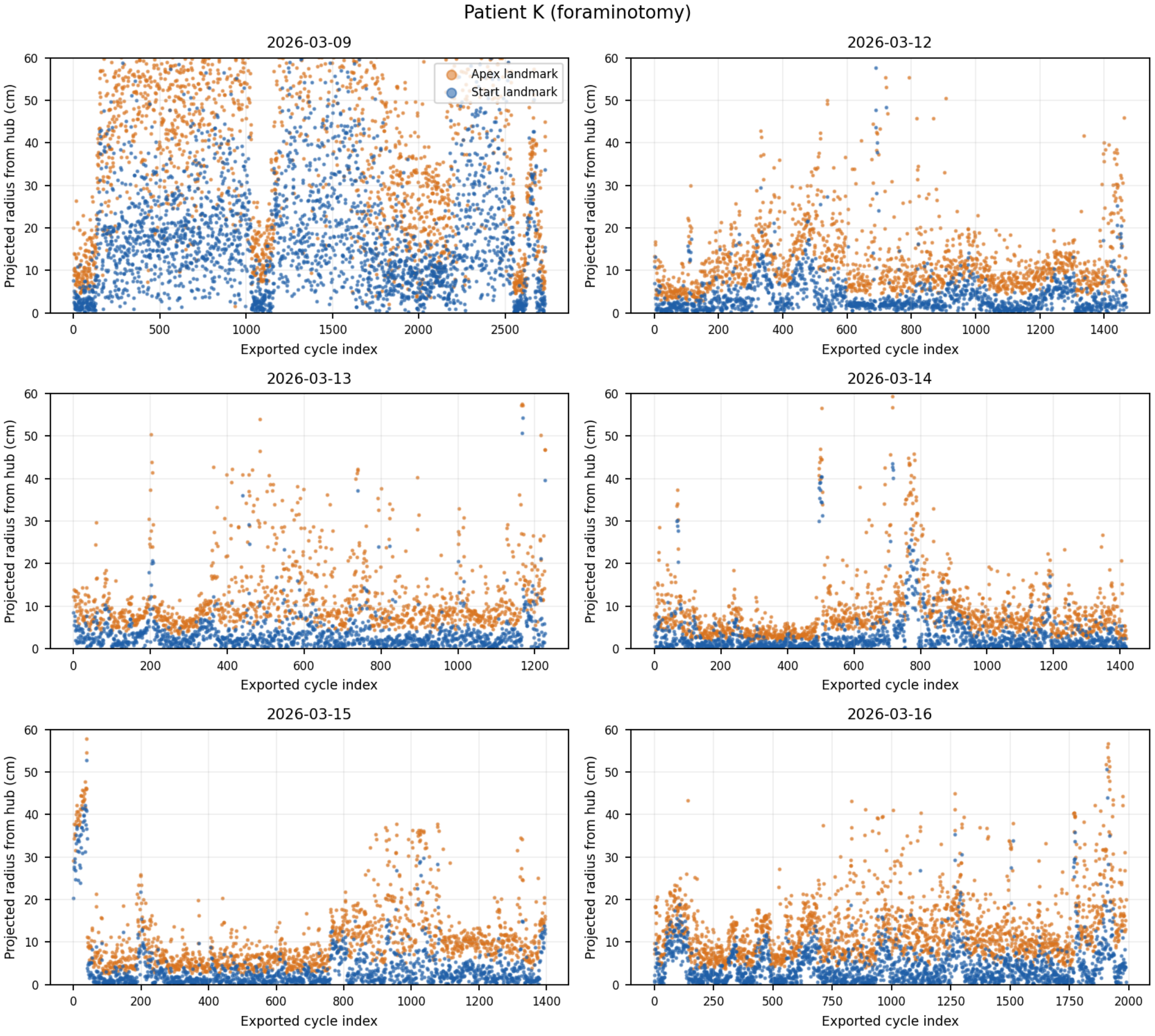

Figure S1a. Patient K, first six recorded days: complete exported start and apex landmarks against exported cycle index, in centimetres of projected radius. The pre-operative day (9 March) is the first panel.

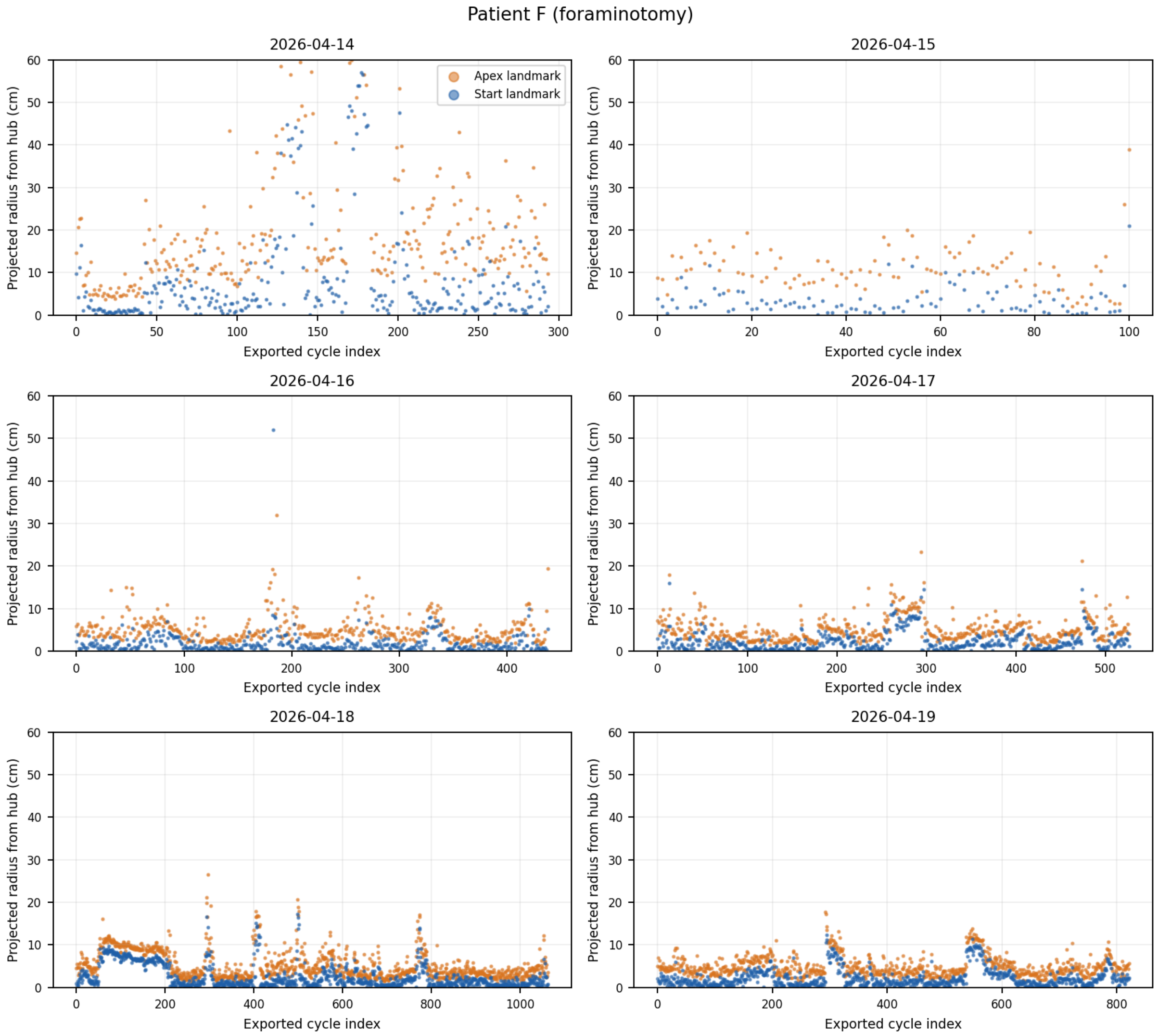

Figure S1b. Patient F, first six recorded days.

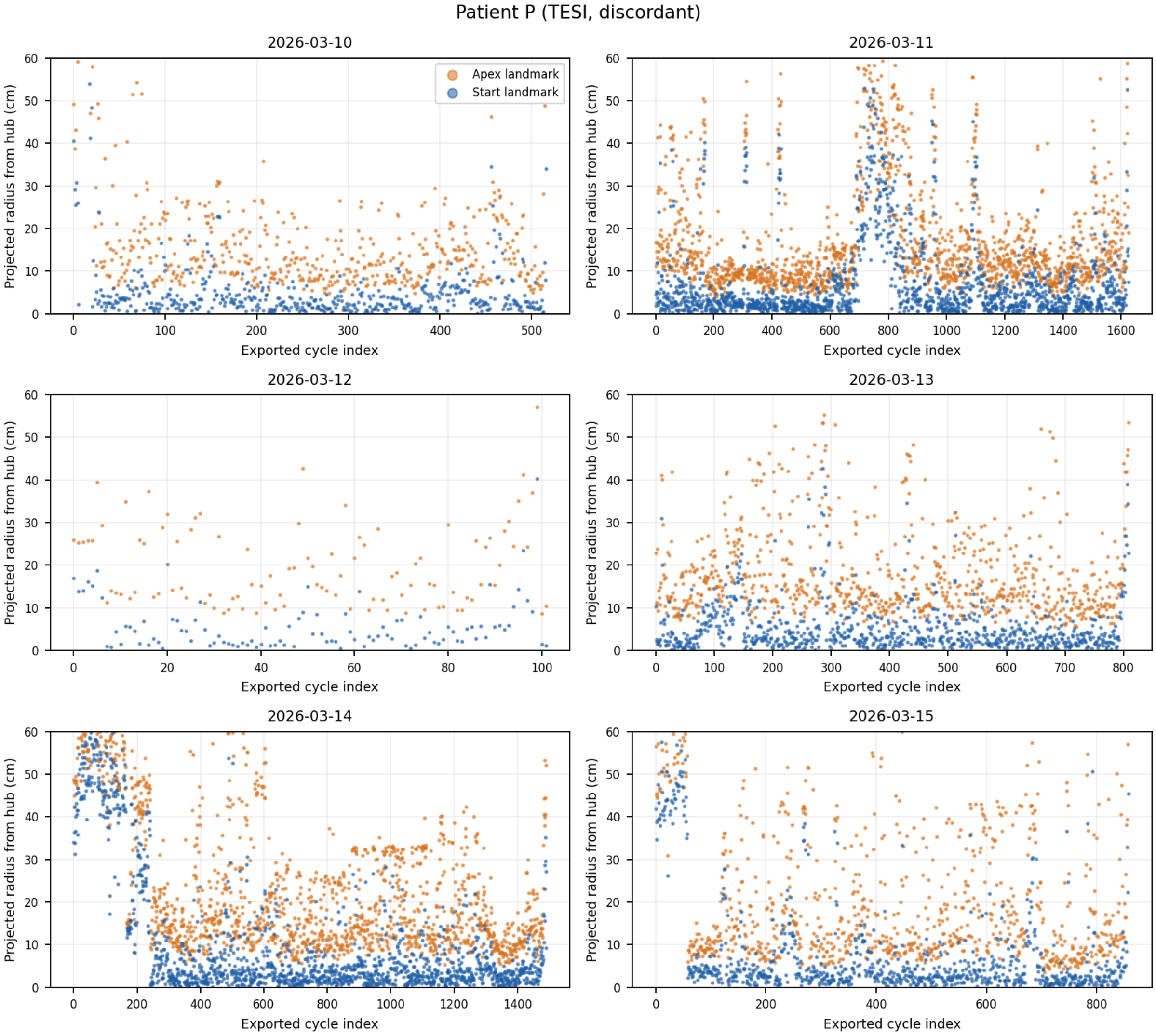

Figure S1c. Patient P (discordant comparison case), first six recorded days.

S5. Secondary activity outputs (descriptive only)

The extractor's heuristic activity classifier produces per-day durations of lying, sitting, standing and walking, sit-to-stand counts, and a walking distance normalised to an assumed stride length. These outputs are unvalidated, their walking and distance masks are not identical, and they are used in this paper only descriptively; they are used as descriptive context only (Section 3.3, Limitations). They are shown because they document that the recordings span ordinary daily behaviour rather than prompted walking.

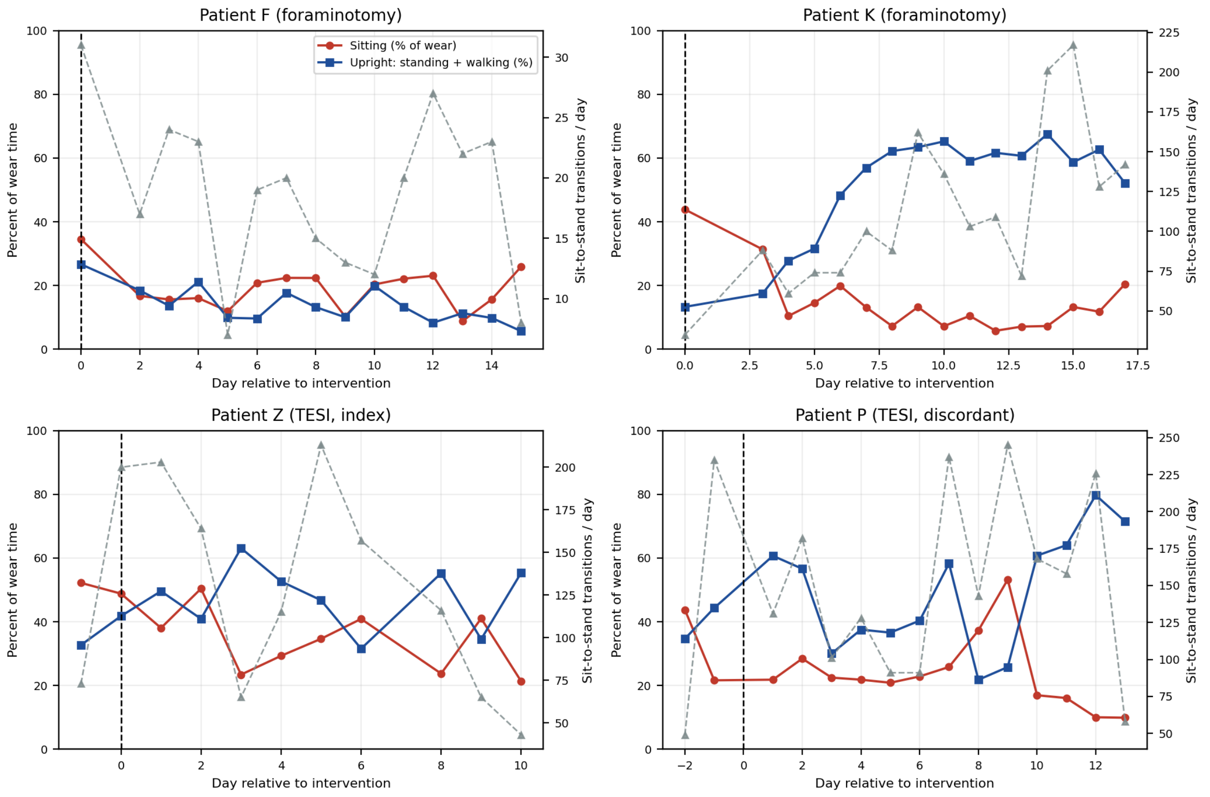

Figure S2. Heuristic classifier outputs per recorded day, per patient: sitting and upright percentages of classified time (left axes) and sit-to-stand transitions (right axes), against day relative to intervention. Descriptive only; the classifier is unvalidated and these curves support no claim in the main text.

Supplementary sensitivity figures

Supplementary tables (CSV)

- **Table S1** (`Table_S1_daily_summaries.csv`): every recorded patient-day with cycle count and the daily apex value under whole-day, first-200, Best-200-apex, Best-200-gap and all-window rules, plus the whole-day start value. 61 rows.
- **Table S2** (`Table_S2_nested_prefix.csv`): Best-200-apex per day and per nested prefix size for the fourteen days with at least 2,000 cycles. 56 rows.
- **Table S3** (`Table_S3_K_leave_one_file_out.csv`): patient K, each pre-operative source file omitted in turn; Cliff's delta on start, apex and apex-minus-start against the full later window. Apex delta ranges 0.70–0.81 and gap delta 0.56–0.65 across omissions; the largest single-file effects come from omitting the two highest-radius files (12:00 and 15:00), consistent with the baseline heterogeneity described in Section 3.4.

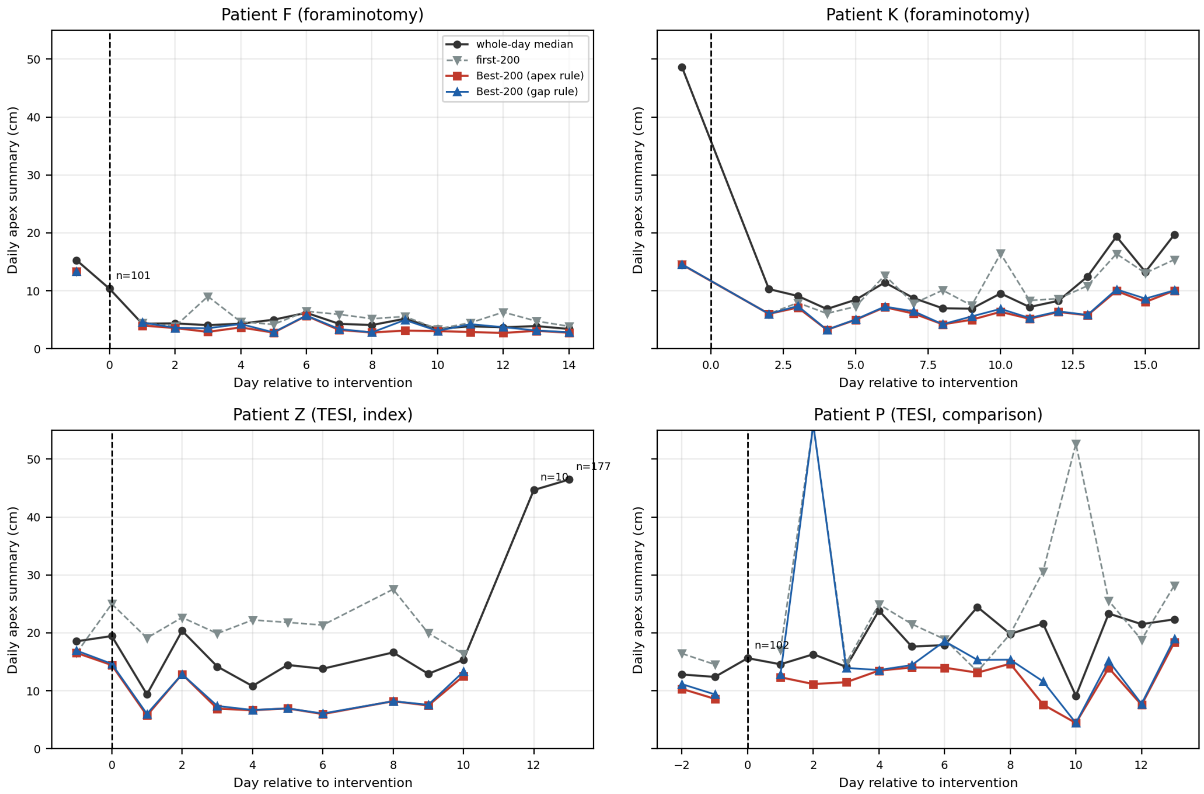

Figure S3. Daily apex value under four summary rules (whole-day median, first-200, Best-200-apex, Best-200-gap) for every recorded patient-day: the whole-day median is shown on all 61 days, including the four low-count dates (annotated with their cycle counts), and the 200-cycle rules where defined. Full values, including the all-window summary, are in Table S1.

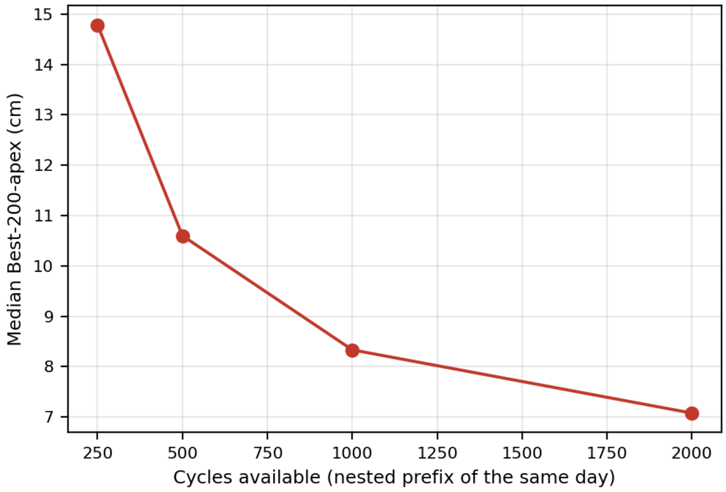

Figure S4. Median Best-200-apex across the fourteen days with at least 2,000 cycles, recomputed on nested prefixes of 250, 500, 1,000 and 2,000 cycles of the same days (Section 3.7 of the main text). The monotone decrease is the expected behaviour of an observed minimum as additional candidate windows are included; it quantifies opportunity dependence and does not establish a physiological lower bound (Section 3.7).
